# Interplay between Diagnostic Criteria and Prognostic Accuracy in Chronic Kidney Disease

**DOI:** 10.1371/journal.pmed.1002129

**Published:** 2016-09-20

**Authors:** Giuseppe Remuzzi, Richard Glassock

**Affiliations:** 1 IRCCS—Istituto di Ricerche Farmacologiche Mario Negri, Bergamo, Italy; 2 Department of Medicine, Unit of Nephrology and Dialysis, Azienda Socio-Sanitaria Territoriale Papa Giovanni XXIII, Bergamo, Italy; 3 Department of Biomedical and Clinical Science, L. Sacco, University of Milan, Milan, Italy; 4 Geffen School of Medicine, University of California, Los Angeles, Los Angeles, California, United States of America

## Abstract

In a Perspective, Giuseppe Remuzzi and Richard Glassock discuss diagnostic criteria for chronic kidney disease and implications for disease progression.

More than a dozen years have elapsed since the original criteria for definition and classification of generic (not specific to type of disease diagnosis) chronic kidney disease (CKD) in adults were first promulgated by the Kidney Disease Outcomes Quality Initiative (KDOQI), sponsored by the United States National Kidney Foundation [1]. The Kidney Disease: Improving Global Outcomes (KDIGO) working group subsequently modified and updated the original classification schema based on a surge in the availability of epidemiological data from large and diverse populations around the globe [2], which included follow-up (of varying durations) for adverse events [3].

The KDIGO schema for generic CKD has been broadly accepted by the nephrology community and has led to the description of CKD as a “common and dangerous” phenomenon with an estimated global prevalence of 11.7%–15.1%, averaging 13.4% in adults [4], but with wide variation between and within countries (3%–25%) [5,6]. Patient and public awareness of CKD, as defined by KDOQI/KDIGO, is generally regarded as very low [7]. It is also widely recognized that CKD, defined in this manner, is largely a disease of older adults. Whether the incidence of CKD is stable, declining, or increasing globally is a matter of heated discussion [8]. The KDOQI and KDIGO schema have been criticized for not taking into account the normal decline in glomerular filtration rate (GFR) with aging [9]. In addition, concerns have arisen over the accuracy of estimating equations for true or measured GFR for diagnosis of CKD [10,11]. Importantly, epidemiological studies using single time-point testing have a built-in propensity for false-positive diagnosis of CKD (which properly includes a time dimension), thus inflating (by 30% or more) the estimated population incidence and prevalence of CKD [12].

Although the epidemiological data have become more detailed and sophisticated, it has remained unclear how this widely advocated schema of diagnosis and prognosis for CKD plays out in the practice of medicine, particularly in primary care. Certainly, widespread adoption of criteria for CKD combined with routine testing and automated reporting of renal function—typically by serum creatinine concentration measurements and estimated GFR (eGFR)—has led to a significant increase in diagnosis of CKD, particularly in the elderly. But how does this newly minted CKD evolve over time? This gap in our knowledge is now filled, at least in part, by a study in this issue of *PLOS Medicine* [13]. Adam Shardlow and colleagues report a novel prospective study of outcomes after 5-year follow-up of 1,741 patients with duration-confirmed stage 3 CKD, managed within 32 primary care practices in Derby, UK, beginning between 2008–2010. The enrolled patients were generally elderly (mean age: 73 years) and predominantly female (60%). The eGFR was estimated using the Modification of Diet in Renal Disease study (MDRD) equation at 53 ± 10 ml/min/1.73 m^2^ at entry. Blood pressures were well controlled in the majority of patients at study entry, and 65% were taking a renin-angiotensin system inhibitor. Progression of CKD was broadly defined as a 25% decline in eGFR coupled with a worsening of GFR category or an increase in albuminuria category. CKD remission was defined as an eGFR >60 ml/min/1.73 m^2^ and a urinary albumin-creatinine ratio (uACR) of <3 mg/mmol (<33 mg/gm) at any study visit, if CKD had been previously diagnosed.

Shardlow and colleagues’ findings are of great interest. At the end of 5 years, 1,237 participants were alive (71%), 247 had died (14%), and 257 had been lost to follow-up or had incomplete data (15%). Of the living participants at risk, stable CKD or remission of CKD was seen in 929 (stable CKD in 34% and remission of CKD in 19%), whereas 308 participants experienced progression of CKD (18%). Remission of CKD at 5 years was independently associated with a higher eGFR at entry, lower age, lower uACR, and a greater increase in eGFR at 1 year. Progression at 5 years was associated with lower eGFR at entry, higher uACR, male sex, and a lower hemoglobin or serum bicarbonate at entry and a greater loss of eGFR at 1 year. Importantly, the enrolled participants showed a low prevalence of proteinuria (average uACR was 0.33 mg/mmol; standard deviation [SD] = 0–1.5 mg/mmol). Also, the observed rate of ESRD over 5 years was very low (<0.2%) but close to the calculated rate (using the four-variable renal failure risk estimator) [14] for an average participant enrolled in the study (0.12%).

Of particular interest was the G3A category of CKD (eGFR of 45–59 ml/min/1.73 m^2^), as this is the most common form of CKD in older adults. As expected, this category of subjects frequently (86%) had no abnormal proteinuria, and <2% had albuminuria (uACR defined) of 300 mg/gm or greater. Among the G3A category without abnormal albuminuria, progression was observed in 17%, but we do not know the relative contribution of a decline in eGFR or an increase in albuminuria to this form of progression. Relevant to these findings, it should be mentioned that a large body of evidence strongly supports the association between proteinuria and risk of CKD progression in both nondiabetic and diabetic patients [15–17].

As the authors carefully acknowledge [13], the findings of this study may not apply to ethnically more diverse or much younger populations. Some degree of self-selection might have occurred among those who agreed to participate in this study, but evidence exists that the cohort examined is reasonably representative of the population of patients with diagnosed CKD in the UK. In addition, it is largely unknown whether the patients with CKD who progressed had underlying diabetes, glomerulonephritis, hereditary disorders, obstructive uropathy, or other conditions.

The study has several additional limitations. First, the eGFR equations include age as a variable. Thus, the relationship of eGFR to mortality is to be expected, as age is a major determinant of the risk of death over any given time period. The measured risks of progression or remission may also be influenced in this study and others by the competing risk of death [18]. Second, although an abnormally increased GFR (above 120 ml/min/1.73 m^2^) in an adult (especially in the presence of diabetes) is a risk factor for future decline in GFR, the impact of “hyperfiltration” (GFR >120–130 ml/min/1.73 m^2^) was not closely examined in this cohort, as eGFR is a rather insensitive method for determining the existence of hyperfiltration, at least in patients with type 1 or 2 diabetes [10,19]. Third, and not surprisingly, the specific details of management principles followed by the primary care physician practices included in this study could not be monitored. Finally, the extent to which the results were influenced by outcome determinants (such as smoking cessation, diet, blood pressure control, or use of potentially nephrotoxic medications) or new-onset comorbidity (such as congestive heart failure, diabetes, or obstructive uropathy), so common in an elderly population, could not be assessed because they were not examined.

Nevertheless, the study and its findings are of great value for furthering our understanding of generic CKD as it occurs in the community at large and within primary care. The study identifies factors, easily obtained and possibly modifiable, that should heighten surveillance for the minority of subjects with CKD stage 3 who are at risk for progression of CKD or cardiovascular disease (CVD). Many of these factors have been recognized previously, especially higher values of uACR or dipstick proteinuria [20]. Most significantly, the study highlights that generic CKD, as defined by KDOQI and KDIGO, is a rather benign condition, even in the older adult, with stability or regression in the majority. It strengthens the case for an age-sensitive redefinition of generic CKD, at least when the diagnosis of CKD is based on eGFR alone (<60 ml/min/1.73 m^2^ in the absence of kidney damage). Such a revision might reduce the unnecessary labelling of older adults as having CKD. The call for a universally accepted definition of what constitutes remission of CKD is also highly appropriate, as such remissions are apparently quite common in older subjects with stage 3 CKD, and this phenomenon deserves more careful attention in the future.

## Abbreviations

CKD: chronic kidney disease
CVD: cardiovascular disease
eGFR: estimated glomerular filtration rate
KDIGO: Kidney Disease: Improving Global Outcomes
KDOQI: Kidney Disease Outcomes Quality Initiative
MDRD: Modification of Diet in Renal Disease study
SD: standard deviation
uACR: urinary albumin-creatinine ratio
